# Genetic Diversities and Historical Dynamics of Native Ethiopian Horse Populations (*Equus caballus*) Inferred from Mitochondrial DNA Polymorphisms

**DOI:** 10.3390/genes12020155

**Published:** 2021-01-25

**Authors:** Kefena Effa, Sonia Rosenbom, Jianlin Han, Tadelle Dessie, Albano Beja-Pereira

**Affiliations:** 1 Ethiopian Institute of Agricultural Research (EIAR), Addis Ababa P.O. Box 2003, Ethiopia; 2 Research Center in Biodiversity and Genetic Resources (CIBIO), University of Porto, 4485-661 Vairao, Portugal; sprosenbom@gmail.com (S.R.); albanobp@fc.up.pt (A.B.-P.); 3 CAAS-ILRI Joint Laboratory on Livestock and Forage Genetic Resources (JLLFGR), Institute of Animal Science, Chinese Academy of Agricultural Sciences (CAAS), Beijing 100193, China; h.jianlin@cgiar.org; 4 International Livestock Research Institute (ILRI), Addis Ababa P.O. Box 5689, Ethiopia; t.dessie@cgiar.org; 5 DGAOT, Faculty of Sciences, University of Porto, 4485-661 Vila do Conde, Portugal

**Keywords:** d-loop sequence, haplotype, genetic variation, feral horse, *Equus caballus*

## Abstract

Matrilineal genetic diversity and relationship were investigated among eight morphologically identified native Ethiopian horse populations using polymorphisms in 46 mtDNA D-loop sequences (454 base pairs). The horse populations identified were Abyssinian, Bale, Borana, Horro, Kafa, Kundido feral horses, Ogaden and Selale. Mitochondrial DNA D-loop sequences were characterized by 15 variable sites that defined five different haplotypes. All genetic diversity estimates, including Reynolds’ linearized genetic distance, genetic differentiation (*F*_ST_) and nucleotide sequence divergence (*D*_A_), revealed a low genetic differentiation in native Ethiopian horse populations. However, Kundido feral and Borana domestic horses were slightly diverged from the rest of the Ethiopian horse populations. We also tried to shed some light on the matrilineal genetic root of native Ethiopian horses from a network constructed by combining newly generated haplotypes and reference haplotypes deposited in the GenBank for Eurasian type Turkish Anatolian horses that were used as a genetic conduit between Eurasian and African horse populations. Ninety-two haplotypes were generated from the combined Ethio-Eurasian mtDNA D-loop sequences. A network reconstructed from the combined haplotypes using Median-Joining algorithm showed that haplotypes generated from native Ethiopian horses formed separate clusters. The present result encourages further investigation of the genetic origin of native African horses by retrieving additional mtDNA sequences deposited in the GenBank for African and Eurasian type horses.

## 1. Introduction

Review of the history of animal domestication shows that since the domestication of horses and donkeys around 4000 and 3000 BC, respectively, no large mammals have been added to our repertoire of successful domesticates. Scholars strongly argue that these two species of the genus *Equus* were the last of the common Old World livestock to be domesticated [1,2]. Though it is confirmed with certainty that domestic donkeys were an African domesticate [3], the center of domestication of domestic horses remained enigmatic [4,5].

Likewise, the history and genetic origin of African horses are contentious. Earlier continent-wide exploration of the domesticated animals of Africa by Epstein [6] and later assessment of the horses of the world by Goodal [7]) show that it is not known with certainty if Africa ever had any indigenous breeds of horses. Most scholars believe that horses were initially introduced into the African continent via Egypt, probably interbred with those native African horse populations and possibly diversified into different racial types in Africa. With the current genetic studies uncovering multiple domestication events in horses [4,5], the possibility that Africa had its own indigenous domestic horses cannot be completely ruled out.

Several historians and geneticists [1,8] who studied the evolution of horses amply described that horses played pivotal roles in the course of human civilizations. In human history, no other animals have played such a direct role in accelerating social processes and political developments as the horse; it has been central to the rise and fall of empires and the conquest of entire continents. They may have enabled herdsmen speaking the first Indo-European languages to begin the expansion that would eventually stamp their languages on much of the world [1]. Jared Diamond [1] who studied world history in the past 13,000 years revealed that domestication of horses revolutionized the world in a way that no other animal ever rivaled. A few millennia later, hitched to battle chariots, horses became the unstoppable jeeps and Sherman tanks of ancient war in Europe. They enabled Attila the Hun to devastate the Roman Empire, Genghis Khan to conquer an empire from Russia to China, and military kingdoms to arise in West Africa. During the colonial period, a few dozen horses helped the Spanish conquistadors, Cortes and Pizarro, leading only a few hundred Spaniards each, to overthrow the two most populous and advanced New World states, the Aztec and Inca empires in South America. With futile Polish cavalry charges against Hitler’s invading armies in September 1939, however, the military importance of this most universally prized of all domestic animals finally seemed to come to an end after 6000 years.

However, recent trends have shown that humans need to maintain their relationships with horses even in the epoch of motorized vehicles, tractors and planes, if not for other reasons, for their power, agility, gracefulness, romantic traditions, sports and leisure [8,9]. For instance, horse sports became part of the popular Olympic Games in the Stockholm Olympics in 1956.

**Figure 1:**
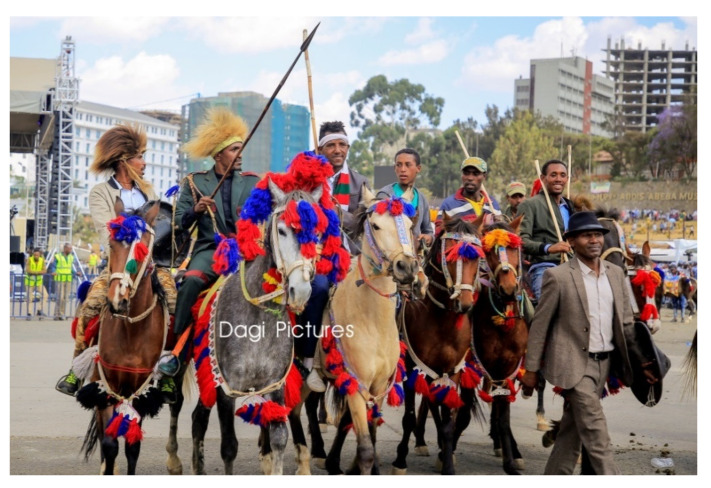
Horse parade to celebrate the 123rd anniversary of Adwa victory as led by prominent Ethiopian musician Hachalu Hundessa.

Likewise, horses played significant roles in the course of Ethiopian civilizations. For instance, a characteristic feature of the traditional Ethiopian social scene was the custom of calling chiefs, warriors and other persons of status after the names of the horses they rode [10]. Such traditional calling was introduced by Oromo tribe who used horses in great battles [10]. Horses made a marked shift in the course of Ethiopian history during the European colonization of Africa where Ethiopian horsemen and patriots defeated a well-armed European power at the battle of Adwa on 2 March 1896 as recently demonstrated in the parade organized by the late prominent Oromo singer Hachalu Hundessa at Meskel Square in Addis Ababa on 2 March 2019 (Figure 1). Today, horses and donkey are essential assets to agriculture and remain sole means of land transport systems in much of the rural communities in Ethiopia.

Despite such exciting stories and anecdotal information collected during this study, the genetic diversity, history and origin of Ethiopian horses have never been adequately documented in the Ethiopian historical chronicles. It is only for the first time that [11] assessed the existence of seven morphologically distinct domestic and one newly explored feral horse populations in Ethiopia.

Therefore, there were multiple objectives of this study: (1) to assess mtDNA genetic diversity and discern genetic relationship between morphologically identified native Ethiopian horse populations; (2) to assess the matrilineal genetic relationship between native Ethiopian and Eurasian type Turkish Anatolian horses that were used as a genetic conduit between Eurasian and African horses; and (3) to augment previous morphological characterization of Ethiopian horse populations with genetic data.

## 2. Materials and Methods

**Figure 2:**
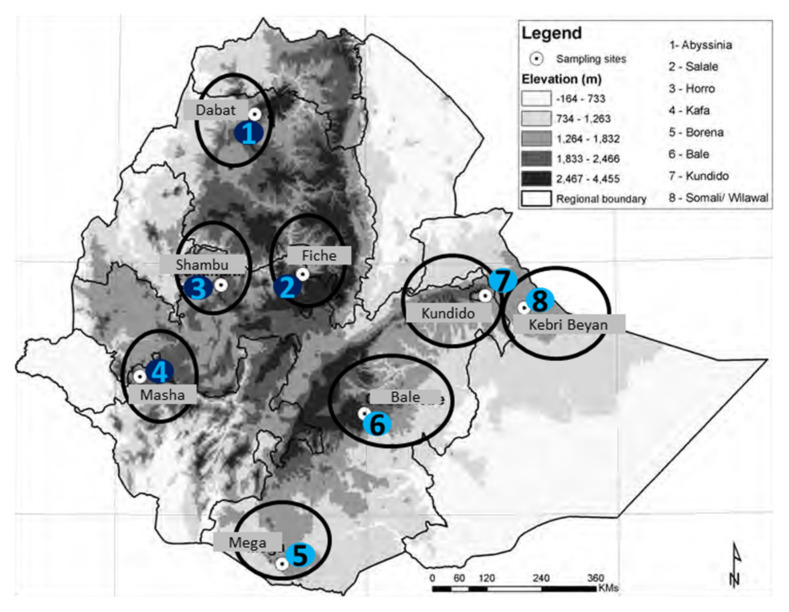
Sampling sites and horse populations represented.

Sampling strategies and sampling sites are as indicated in Figure 2 and Table 1.

Whole blood samples were collected from a total of 46 genetically unrelated adult horses that belonged to seven phenotypically identified domestic and one newly explored feral horse populations. Blood samples were collected by puncturing a jugular vein followed by quick dropping of the blood on Whatman^®^ FTA Classic Card following manufactures instructions. The number of samples per breed and sex of animals are indicated in Table 2. Collected samples were stored at room temperature before to be shipped to CIBIO (Research Center in Biodiversity and Genetic Resources), Universidade do Porto, Portugal) for DNA extraction and further genetic analysis. All applicable international, national and/or institutional guidelines for the care and use of animals were strictly followed.

Genomic DNA was extracted using JETQUICK Blood and Cell Culture DNA Spin Kit with some modification made to manufacture’s protocol (Supplementary Table S1). Polymerase chain reaction (PCR) was carried out in 25 µL reaction volumes containing 4 µL of genomic DNA, 2.2 mM of MgCl_2_, 0.4 mM of each ddNTP, 1 µM of each primer, 0.4 U/tube Platinum *Taq* (Platinum, Invitrogen) and 0.25 µg/µL BSA. Amplification of the D-loop region (454 bp) was done using the oligonucleotide primers (DONK_F: CCCAAGGACTATCAAGGAAG for forward primer and DONK_R: GGAATGGCCCTGAAGAAAG for reverse primer) as described in Beja-Pereira et al. (2004). PCR was carried out using GenAmp 9700 (Applied Biosystems) with the following conditions: initial denaturation at 94 °C for 15 min followed by 45 cycles of denaturation each at 94 °C for 1 min, annealing/hybridization at 56 °C for 1 min, extension at 72 °C for 1 min, and a final extension at 72 °C for 20 min.

PCR products were purified and sequenced for both strands at the High-Throughput Genomics Unit (HTGU), Department of Genome Sciences, University of Washington (http://www.htseq.org/). The raw sequence trace files were aligned and checked using software package DNASTAR v7.1 (DNASTAR Inc., Madison, WI, USA). The resulting mtDNA D-loop sequences were edited for the presence of any ambiguous bases using SeqMan by aligning with standard mtDNA D-loop sequences deposited in the GenBank (accession nos. AF132568-AF132594). Multiple alignments were performed using Clustal W [12] in MegaAlign with default gap penalties and the resulting alignments were verified by eye. Both computer software programs are available in DNASTAR v7.1 (DNASTAR Inc. Madison, WI, USA). The final sequence lengths were edited to 454 nucleotides and the resulting sequences were deposited in the GenBank (accession nos: JX673983-JX674028).

The second dataset of homologous sequences, which were used to trace the matrilineal genetic root of Ethiopian horses, were retrieved from the GenBank (accession nos. HM483870-HM484172) deposited for Anatolian Turkish horses. All the 302 mtDNA D-loop sequences for Turkish Anatolian horses and the new sequences from 46 Ethiopian domestic horses (including six sequences from *Kundido* feral horses) were truncated back to 267 matching base pairs. Sequence similarity search was done with DNASTAR v7.1. Maternal genetic signature of Ethiopian horses was assessed from haplotypes generated from the combined sequences via Median-Joining network analysis performed in NETWORK v. 4.6 [13]. In both datasets, reticulations were resolved through maximum parsimony (MP) criteria where non-MP links were deleted from the network. A simple justification for using mtDNA D-loop sequences from Turkish Anatolian horses for genetic lineage comparison was that horses from this region of Turkey were widely believed to be used as a genetic conduit between European and Asian horses [14] before they were introduced to Africa.

### Data Analysis

Assuming short divergence times between Ethiopian horse populations, Slatkin’s linearized genetic distances [15] were computed using ARLEQUIN v 3.5.1.2 [16]. The statistical significance of the values was estimated by permutation analysis using 1000 replications on the basis of uniform substitution rates among sites. Clustering analysis using the neighbor-joining method [17] was performed in MEGA 5.0 software package [18]. The robustness of the resulting dendrogram was checked by running 1000 bootstrap replicates.

Extent of mtDNA polymorphisms such as haplotypes (gene) diversities (Hd), nucleotide diversities (π) and average number of nucleotide differences (*K*) [19,20], number of segregating sites and number of haplotypes per population were calculated using DnaSP5 v. 5.10.01 [21]. Network from the combined haplotypes was reconstructed using Median-Joining network in NETWORK v. 4.6 software package [13]). Moreover, the extent of population genetic differentiations was tested using DnaSP5 software package [21] with bootstrap replicates set at 10,000. Sites that contain alignment gaps and missing information had been completely deleted before the analysis begun.

## 3. Results

### 3.1. Haplotype Diversities in Native Ethiopian Horse Populations

**Figure 3:**
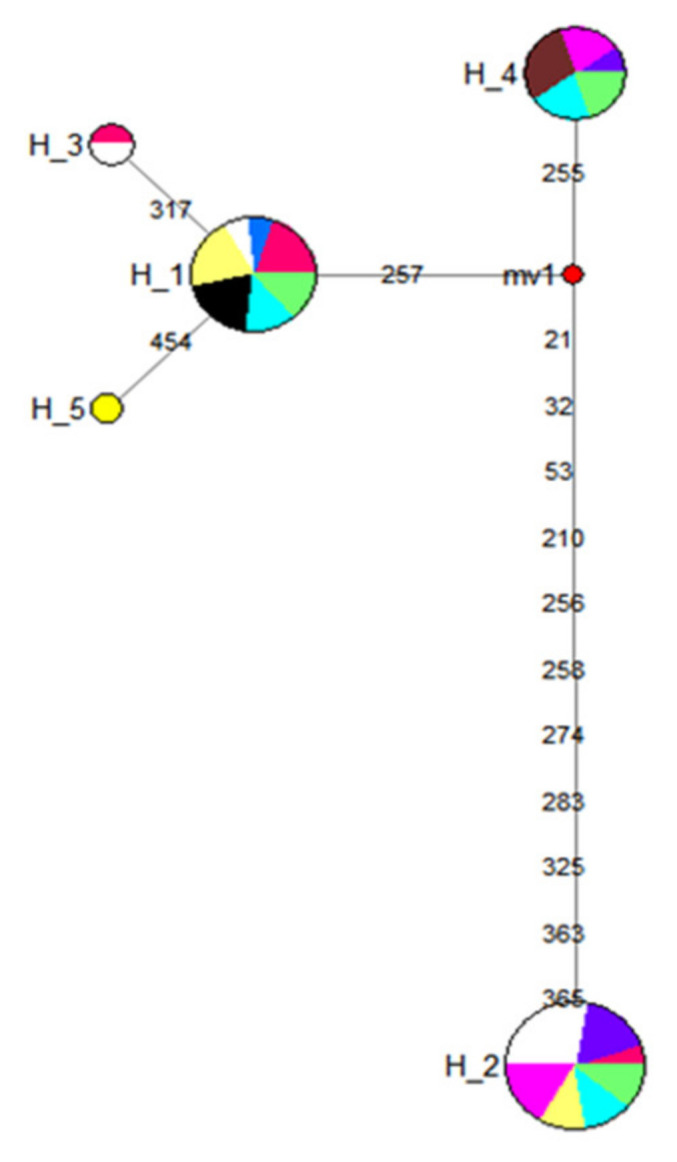
Median-Joining network of native Ethiopian horses (Abyssinian = Red; Bale = Blue; Boran = White; Horro = Pink; Kafa = Yellow; Kundido = Black; Ogaden = Magenta; and Selale = Green). Circles are proportional to the frequency of individual haplotypes. Median vector is indicated in red. Numbers indicate the number of mutation steps between haplotypes.

The 46 mtDNA sequences in domestic and Kundido feral horse populations were defined by 15 polymorphic sites that further collapsed into five haplotypes (Figure 3), among which were two minor haplotypes, for instance, H5 was unique to only the Kafa horse population while H3 consisted of haplotypes derived from one Abyssinian and another Boran horses. Fewer polymorphic sites that led to the generation of limited number of haplotypes suggested that Ethiopian horses were characterized by narrow matrilineal genetic diversity.

### 3.2. Nucleotide Diversities in Native Ethiopian Horse Populations

Nucleotide diversities for Ethiopian horse populations are presented in Table 3. Average haplotype diversity was estimated to be 0.706 ± 0.032 and ranged from 0.333 in Boran to 0.800 in Abyssinian, Ogaden and Selale horse populations.

Average nucleotide diversities was estimated to be 0.014 ± 0.001 (ranged from 0.0026 in Kundido feral to 0.0167 in Abyssinian horse populations). As in the case of haplotype diversity, Boran horses also demonstrated the second lowest nucleotide diversity among Ethiopian horse populations. The average number of nucleotide differences was 6.320 (ranged from 1.200 in Kundido feral horses to 7.600 in Abyssinian horses). Generally, Boran domestic horses and Kundido feral horses were characterized by low mtDNA diversities while Abyssinian domestic horses possessed the highest mtDNA diversities (Table 3). Interestingly, besides the morphological resemblances observed during field survey, Selale and Ogaden domestic horses had identical mtDNA genetic diversity estimates regardless of the contrasting environments they dwell in (Table 3).

### 3.3. Genetic Distances between Native Ethiopian Horse Populations

Estimates of matrix of linearized *F*_ST_ values between domestic and feral Ethiopian horse populations are shown in Table 4. The result showed that the newly discovered Kundido feral horses were genetically unique and consistently distant from all domestic horse populations except with Abyssinian domestic horses. However, most of the domestic horse populations had narrow genetic bases. For instance, linearized Slatkin’s mtDNA differentiation between Boran and Kundido feral horses (2.734) was the highest followed by those between Kundido feral horses and Bale (0.863) and Kundido feral horses and Horro (0.5000) domestic horses. Abyssinian, Kafa, Ogaden and Selale horse populations were genetically indistinguishable from each other as compared with the rest of the Ethiopian horse populations. Furthermore, within-population genetic distances revealed high genetic diversity in Bale and Horro domestic horse populations. However, Kundido feral horses were characterized by low within-population genetic diversity (Table 4).

**Figure 4:**
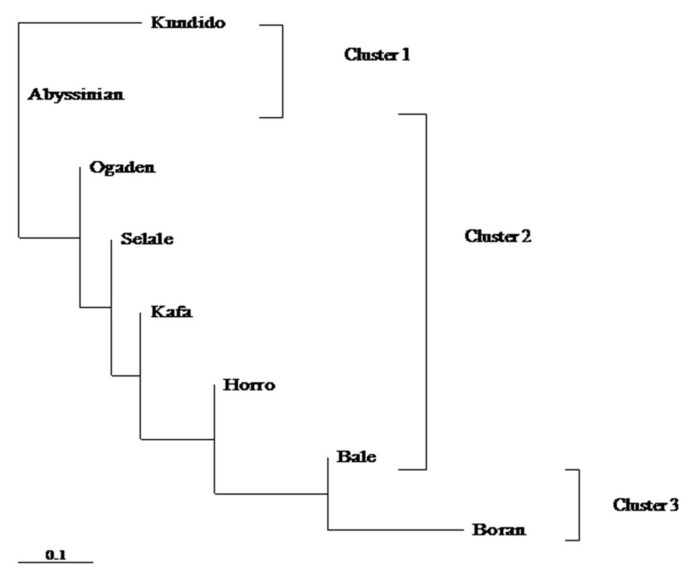
Unrooted neighbor-joining dendrogram constructed from Slatkin’s linearized genetic distances [15]) and Kimura 2-parameter model.

An unrooted network tree constructed using distance-based neighbor-joining algorithm illustrated similar patterns of matrilineal genetic structuring among native Ethiopian horse populations (Figure 4). However, Kundido feral horses formed a slightly separate cluster together with Abyssinian domestic horse population. A dendrogram tree reconstructed from Slaktin’s linearized genetic distances roughly grouped the overall native Ethiopian horse populations into three major genetic clusters: (1) Abyssinian domestic and Kundido feral horses into cluster 1; (2) Bale, Ogaden, Selale, Kafa and Horro into cluster 2; and (3) Borana horses into genetic cluster 3. It is important to note that Abyssinian horses formed mid-point rooting and failed to form any distinct cluster between Kundido feral horses and native horses under cluster 2.

### 3.4. Nucleotide Sequence Divergence and Genetic Differentiation

Results of pair-wise genetic differentiation (*F*_ST_) and nucleotide divergences (*D*_A_) are shown in Table 5. These two classical population differentiation measures also supported low genetic differentiations and nucleotide sequence divergences in both feral and domestic Ethiopian horse populations. Yet, Kundido feral horses showed consistent divergence from the rest of native Ethiopian horse populations. The highest genetic differentiation (76.8%) was observed between Borana domestic and Kundido feral horses followed by that between Borana and Abyssinian (49.7%). Moreover, the two highly diverging populations (Boran domestic and Kundido feral horse) were distant from each other (43.4%). Therefore, Boran domestic horses that inhabited the arid and semi-arid climate of southern rangelands of Ethiopia were the only domestic horse population that diverged the most from the rest of Ethiopian horses followed by Kundido feral horses. The rest of the native Ethiopian horse populations consistently demonstrated low maternal genetic differentiations. Likewise, estimates of pair-wise nucleotide sequence divergences (*D*_A_) were very low between the rest of Ethiopian horse populations while Boran domestic and Kundido feral horses maintained their divergence (Table 5).

### 3.5. Matrilineal Genetic Lineage of Native Ethiopian Horse Populations

**Figure 5:**
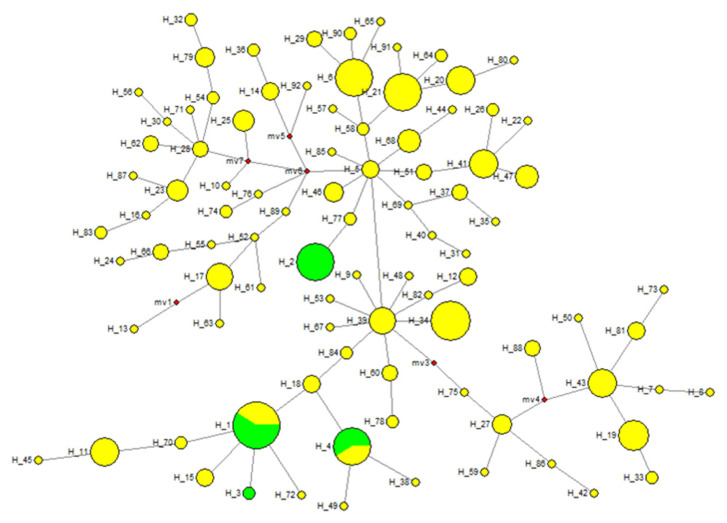
Median-Joining network reconstructed from 92 combined Ethiopian and Eurasian type Turkish Anatolian horse haplotypes (Ethiopian haplotypes = green; Turkish Anatolian haplotypes = yellow). Circles are proportional to the frequency of individual haplotypes.

With the current mtDNA D-loop sequences dataset we used, we assessed the matrilineal genetic root of native Ethiopian horse populations by combining the newly sequenced dataset with the previously published sequences retrieved from the GenBank deposited for Eurasian type Turkish Anatolian horses (HM483870-HM484172). Both datasets were truncated back to 267 matching base pairs. A total of 92 different haplotypes were generated from the combined Ethio-Eurasian horse mtDNA sequences. A network reconstructed from combined haplotypes showed that haplotype H5 and H39, which were solely derived from Eurasian type horses, formed the major and minor centers of the network, respectively (Figure 5). Besides, both centers of the network formed star-like patterns (yellow circles), suggesting wider matrilineal genetic diversities and extensive differentiation into various racial types. The two haplotypes (H2 and H3) were unique to sequences generated from native Ethiopian horses (green circles). Moreover, H1 and H4 were dominated by haplotypes generated from native Ethiopian horses with insignificant matrilineal genetic integration from Eurasian type Turkish Anatolian horses. Unlike the large dataset previously used by Vila et al. (2001), the small dataset we currently analyzed also supported continental mtDNA sequence groupings in domestic horses.

## 4. Discussion and Conclusions

### 4.1. Haplotype Diversities in Native Ethiopian Horse Populations

According to the comprehensive guidelines developed by FAO (2011), characterization of farm animal genetic resources for food and agriculture (AnGR) involves three types of information: phenotypic, molecular and historical. The weight given to each depends on the country (i.e., whether it is developed or developing) and objectives (e.g., improvement, conservation or breed differentiation). In our previous study, we used FAO (2011) guidelines to characterize the morphological traits and geographical distributions of Ethiopian equines in which we reported the existence of seven morphologically unique and one feral horse population [11]. However, this study has not been supported by genetic data to assert whether the diverse morphological variations observed are concordant with the underlying genetic background.

Our present study revealed that the 446 bp long mtDNA D-loop sequences in Ethiopian horses were characterized by fewer polymorphic sites and the resulting haplotypes generated from these sequences showed high matrilineal genetic admixture except for Kafa horses. This showed that Ethiopian horse populations had a narrow maternal genetic basis regardless of observable morphological differences and dissimilar ecozones they dwell in.

### 4.2. Genetic Diversities and Historical Connections in Native Ethiopian Horse Populations

During this extensive and exploratory type survey, the first author travelled across all over Ethiopia where horses are commonly reared and conducted personal interviews with local elders to complement the study with anecdotal information and historical backgrounds (data not presented).

It is interesting to note that the genetic divergences of Kundido feral horses and Borana domestic horses from the rest of Ethiopian horse population are concordant with the morphological variation reported in [11]. The home tract of Kundido feral horses is the high rising Kundido Mountain plateau in eastern Ethiopia, which has never been inhabited by any other horse population. Their presence on this high rising mountain plateau is mysterious. As there is no documented information on this population, it is not known with certainty when, how and why Kundido feral horses went into the feral state. From our understanding of the feral and domestic horses of Africa, the Kundido feral horses are perhaps the oldest feral horse populations living in a wild state in Africa. Following our exploration and report, they gained popularity and are currently are under conservation and preservation program by Oromia Environment and Wildlife Authority of the Federal Democratic Republic of Ethiopia.

Likewise, the graceful Borana domestic horses are maintained by the famous Borana pastoral community near Mega Town in the arid and semi-arid ecosystem of Southern Borana Rangelands of Ethiopia. Local pastoralists reported that the population size of Borana horses is rapidly declining over time due to recurrent drought and increasing aridity in the pastoral ecosystem. Like Kundido feral horses, they warrant an urgent conservation program.

From a classical genetics viewpoint, genetic divergences between Kundido feral and Borana horses and the rest of the Ethiopian horse population can be explained by geographical/population isolation as they dwell in isolated ecosytems as compared to other horse populations. Furthermore, adaptation processes to local climate, cultural separation and genetic drift might be some of the driving forces for morphological and genetic distinctiveness of these two populations.

The value greater than 1 *F*_ST_ obtained using Slatikn’s linearized genetic distances between Borana domestic and Kundido feral horses might be due to the fact that the linearized Slatkin’s *F*_ST_ method utilizes a stepwise mutation model in the estimation procedure, which probably resulted in a higher *F*_ST_ value.

Interestingly, classification of native Ethiopian horses into eight phenotypic groups is incongruent with molecular data. Hence, differences in morphological character system within a species cannot guarantee populations with unique morphological characteristics are also genetically distinct. Such phenotypic differences may implicate adaptive divergences of a given population to a prevailing environment. This agrees well with the findings reported in [22] who elucidated that differences in morphological character systems reflect ecological selection regimes (coat color patterns), history (body dimension) or both (scalation) and may or may not support the variation at a molecular level.

Sometimes, morphological and molecular variations and/or similarities at population level have to be carefully interpreted and if possible it has to be supported by population history. Identical haplotype, nucleotide and average number of nucleotide difference estimates observed between Selale and Ogaden horses were one of the interesting findings that attracted our attention. The result highly concurs with some anecdotal stories told by local Somali elders who stated that the former Ethiopian King Emperior Hailesillasie had been regularly attending horse show in Somali region and was regularly recruiting some horses from the region to improve the horse stud he established in Selale areas in central Shoa region. However, such anecdotal stories and speculations need to be further augmented with genetic, anthropological and historical connections to prove with certainty that Ogaden and Selale horses have common maternal lineage.

Mitochondrial DNA diversities observed in our present study are generally lower than any other studies reported from Eurasia, central and Latin American countries. For instance, the 0.91 average haplotype diversity, 67 polymorphic sites and 17 different haplotypes reported in 65 Cheju horses sequenced for 968 mtDNA sequences in South Korea [23] is by far higher than the present study. Lopez et al. (2005) [24] also sequenced 408 mtDNA nucleotides in 145 Portuguese Lusitano mares and found about 43 polymorphic sites that produced 27 different haplotypes. Likewise, [25] also sequenced 324 mtDNA D-loop fragments from 55 Croatian horses and found about 26 polymorphic sites that produced 30 different haplotypes. In another study [14] sequenced about 301 nucleotides from 302 Turkish Anatolian horses and identified 58 polymorphic sites that produced 100 different haplotypes. Nucleotide and haplotype diversity obtained from the same Turkish samples are also higher than the results obtained in the present study. This demonstrates that native Ethiopian horses are characterized by relatively low matrilineal genetic diversity.

### 4.3. Matrilineal Genetic Root of Native Ethiopian Horse Populations

It is interesting to observe that haplotypes generated from the combined Ethiopian and Eurasian type Turkish Anatolian horses formed a star-like branching where major haplotypes derived from Ethiopian horses formed unique clusters. Likewise, haplotypes derived from Turkish Anatolian horses formed separate cluster suggesting the fact that Ethiopian and Turkish Anatolian horses have independent material lineages. The present result agrees with the reports of [5] and [4] who reported multiple origination and domestication events in domestic horses. Therefore, our present findings leave some room and pose further research questions on the genetic roots of the horses of Ethiopia in particular and Africa at large, to come up with conclusive evidence as to whether Africa ever had its own indigenous horse breeds independently domesticated in the continent.

## Figures and Tables

**Table 1:** Sampling sites and horse populations represented.

No	Sampling Site	Horse Population Represented
1	Dabat	Abyssinia
2	Goba	Bale
3	Mega	Borana
4	Shambu	Horro
5	Masha	Kafa
6	Kundido	Kundido
7	Kebri Bayan	Ogaden
8	Fiche	Selale

**Table 2:** Population group and number of samples genotyped by sex.

No	Population Name	No. of Samples	Gender	
			M	F
1	Abyssinia	5	0	5
2	Bale	5	3	2
3	Borana	6	0	6
4	Horro	6	2	4
5	Kafa	6	3	3
6	Kundido	6	3	3
7	Ogaden	6	6	0
8	Selale	6	5	1
		46	22	24

**Table 3:** Horse populations, sample sizes (n), number of polymorphic sites (S), haplotypes per population (Hp), haplotype diversity (Hd), nucleotide diversity (π) (Nei, 1987) and average number of nucleotide differences (*K*) and their standard deviations in native Ethiopian horse populations.

Population	n	S	Hp	Hd ± S.D.	π ± S.D.	*K*
Abyssinian	5	13	3	0.800 ± 0.164	0.017 ± 0.005	7.600
Bale	5	13	3	0.700 ± 0.218	0.016 ± 0.005	7.400
Boran	6	13	2	0.333 ± 0.215	0.010 ± 0.006	4.330
Horro	6	13	3	0.733 ± 0.155	0.017 ± 0.003	7.467
Kafa	6	13	3	0.733 ± 0.155	0.015 ± 0.004	6.733
Kundido feral	6	2	2	0.600 ± 0.129	0.003 ± 0.001	1.200
Ogaden	6	13	3	0.800 ± 0.122	0.015 ± 0.004	6.933
Selale	6	13	3	0.800 ± 0.122	0.015 ± 0.004	6.933
Average				0.706 ± 0.032	0.014 ± 0.001	6.320

**Table 4:** Matrix of Slatkin’s linearized genetic distances (*F*_ST_) between native Ethiopian horse populations.

Population	Abyssinian	Bale	Boran	Horro	Kafa	Kundido Feral	Ogaden	Selale
Abyssinian	0							
Bale	0.149	0						
Boran	0.837	0.000	0					
Horro	0.055	0.000	0.064	0				
Kafa	0.000	0.000	0.385	0.000	0			
Kundido feral	0.092	0.863	2.734	0.5000	0.218	0		
Ogaden	0.000	0.000	0.351	0.000	0.000	0.148	0	
Selale	0.000	0.000	0.351	0.000	0.000	0.148	0.000	0
Within population	0.011	0.016	0.010	0.016	0.015	0.003	0.015	0.015

**Table 5:** Matrix of pair-wise Hudson et al. (1992b) *F*_ST_ (below diagonal) and *D*_A_ distances (D-loop nucleotide sequence divergences, above diagonal) between seven domestic and one feral Ethiopian horse populations.

Population	Abyssinian	Bale	Boran	Horro	Kafa	Kundido Feral	Ogaden	Selale
Abyssinian	-	0.002	0.009	0.001	0	0.000	0	0
Bale	0.130	-	0	0	0	0.007	0	0
Boran	0.497	0	-	0.001	0.006	0.018	0.005	0.005
Horro	0.059	0	0.104	-	0	0.005	0	0
Kafa	0	0	0.325	0	-	0.002	0	0
Kundido feral	0.059	0.434	0.768	0.333	0.179	-	0.001	0.001
Ogaden	0	0	0.306	0	0	0.129	-	0
Selale	0	0	0.306	0	0	0.129	0	-

## Data Availability

Forty-six mitochondrial DNA sequences edited to 454 nucleotides were deposited in the GenBank, National Center Biotechnology Information (NCBI) with GenBank ID between JX673983-JX674028.

## Supplementary Materials

The following are available online at https://www.mdpi.com/2073-4425/12/2/155/s1, Table S1: Modified laboratory protocol used to extract nuclear DNA from Whatman FTA Classic Cards.
