# Supplementary material for: Genetic Diversities and Historical Dynamics of Native Ethiopian Horse Populations (Equus caballus) Inferred from Mitochondrial DNA Polymorphisms
Source: Genes (Basel). 2021 Jan 25;12(2):155. doi: 10.3390/genes12020155 (PMC7912211; doi:10.3390/genes12020155)
Supplement: Supplementary file 1 [file genes-12-00155-s001.pdf]

Table S1. Modified laboratory protocol used to extract nuclear DNA from Whatman FTA Classic Cards.

I. Purifying DNA from FTA card matrix	Sufficient punches of FTA card matrix that contain immobilized DNA were cut, grinded into pretty small pieces and transferred into 1.5 mL centrifuge tubes. Blades and forceps were sterilized between each sample following standard laboratory procedure. Pieces of FTA cards were first digested using 200 µl of tissue lysis solution (T_1) and 20 µl protease K followed by incubating at 56°C overnight. In the next morning, 200 µl of second tissue lysis solution (T_2) was added, vigorously vortexed and re-incubated at 70°C for 10 minutes. After 10 minutes, the samples were taken out and 200 µl of 95% ethanol stored at 4°C was added to the sample solution and vortexed immediately to avoid DNA sedimentation. Using pipette, the lysate was directly applied to the JETQUICK Micro Spin Column that contains silica membrane and the digestion was discarded.
II. Binding and washing	The JETQUICK Micro Spin Columns that contain lysate were centrifuged at 8000 rpm for one minute and the solution was disposed. The DNA suggested be bound the silica membrane was washed first by adding 500 µl washing solution 1 (T_X) and centrifuged at 8000 rpm for one minute followed by a final washing using 500 µl washing solution 2 (T_3) and centrifuging it at 13,000 rpm for three minutes. These two subsequent washing removed residual contaminants and the DNA was selectively bound to the highly specified silica membrane.
III. Elusion	Finally, DNA bound to surface-specified silica membrane was eluted twice using 60 µl DNA elusion solution (10 mM Tris-HCl) where each elusion was centrifuged at 8000 rpm for two minutes. Stock FTA elutes (120 µl) were stored at 4°C for further downstream applications.
